# Silver linings: how mental health activists can help us navigate wicked problems

**DOI:** 10.1192/bjb.2021.25

**Published:** 2021-08

**Authors:** Neil Armstrong, Keira Pratt-Boyden

**Affiliations:** 1 Magdalen College, University of Oxford, UK; 2 School of Conservation and Anthropology, University of Kent, UK; 3 SOAS University of London, UK

**Keywords:** Climate change, wicked problems, psychiatry, ethnography, mental health service survivors

## Abstract

This article explores how ‘wicked problems’ such as climate change might force psychiatry to rethink some of its fundamental ideas and ways of working, including clinical boundaries, understandings of psychopathology and ways of organising. We use ethnographic evidence to explore how mental health service ‘survivor’ activists are already rethinking some of these issues by therapeutically orienting themselves towards social problems and collective understandings of well-being, rejecting ‘treatment as usual’ approaches to distress. In this way we provide an example of the potential of activists to help psychiatry negotiate the climate crisis.

In *Hyperobjects: Philosophy and Ecology after the End of the World,* Timothy Morton suggests that the climate emergency is a ‘wicked problem.’^1^ Problems might be described as wicked in cases where their complexity, our lack of knowledge or the absence of stable, defined goals make them extremely difficult (or even impossible) to solve. Wicked problems are often highly entangled and interdependent, such that addressing one area leads to unwanted consequences in another. When dealing with a wicked problem, the answer does not lie in a single solution. Wicked problems require broad perspectives, which examine social processes and systems and are responsive to phenomena that may be marginal or poorly understood. Morton argues that the wicked problem of the climate crisis is placing an intense and transformative pressure on our patterns of reasoning and that it demands an overhaul of some of our key ideas and values. As Boulton notes, climate change ‘renders *vulnerability* [our italics] as the tangible human experience of environmental degradation and destabilizes our sense of existence’ to the extent that it challenges ‘human-scale understandings of personhood, planetary existence, and cognition in general’.^2^

We suggest that psychiatry cannot be left out of any overhaul of key ideas or values, not least when they pertain to vulnerability and our understanding of the person. There are at least two aspects to this. First, we suggest that the climate crisis disrupts ideas about psychopathology that draw on notions of autonomy, independence, functioning, goal orientation and economic activity. Second, we note how the crisis unsettles critical but unexamined assumptions about how individuals and institutions should be organised by questioning the values of orderliness, preparedness and managerial rationality often found within organisations such as the UK's National Health Service (NHS) and Department for Work and Pensions. To investigate this we draw on ethnographic fieldwork with mental health service ‘survivor’ activist networks in London, who reject ‘treatment as usual’ and challenge some of the main premises of mental illness and what it means to live healthily. We explore interlocutors’ preferences for ‘dis-organisation’ and disorder in group meetings as a therapeutic act. We also present how activists reframe their inability to work and to receive sickness benefits as a means of community engagement, collective ethics and undertaking social ‘goods’. Ethical approval for the fieldwork cited in this article was approved by the University of Kent. Verbal informed consent was obtained from all participants.

## Mental health service survivor activists: the Challenge Mental Health network

Mental health service survivor activists have iatrogenic and traumatic experiences of NHS mental healthcare. As a collective, they draw their alternative understanding of what it means to be a valuable member of society away from individual ‘functions’ and towards socioenvironmental concerns. Global phenomena such as the climate emergency are used by activists to think through how mental distress is conceptualised in mental health services. They find that it no longer makes sense to work within a framework and discourse of individual responsibility when the climate emergency shows how profoundly interconnected our lives really are. In this article, we provide ethnographic material to think these issues through, suggesting not solutions but ways that psychiatry might begin to respond to the climate crisis in a more inclusive and holistic manner.

Survivor groups such as Challenge Mental Health (CMH) network meet across London to campaign for better understandings of mental distress and improvement of local and national mental health services and treatments. One of CMH's main premises is that psychiatry underestimates the social causes and treatments of ‘mental illness’ (or what they term ‘distress’). They argue that undervaluing social processes in this way contributes to poor mental health. As a group, CMH are opposed to thinking in terms of individual vulnerabilities or pathologies. Instead, they emphasise social attitudes, collective pathologies and systemic barriers that impede recovery. They concentrate on collectively alleviating social influences of distress and support one another by telling personal stories of distress and discussing ideas around illness beliefs and causes. They also allow individuals to experience distress in the presence of the group without intervention or treatment.

The way survivor groups organise themselves reflects an ambivalent relationship with bureaucratic working; this in part involves a conscious construction of a meeting space which is entirely ‘other’ from places such as the clinic or the hospital. At first glance, these preferences might appear at odds to those with training in goal-oriented therapeutic approaches. CMH have particular ways of approaching their meetings for example. They reject orderly, rule-governed practices and behaviours which are rooted in planning and preparedness and which might remind them of NHS ‘treatment as usual’. CMH meet according to consensus in spaces all over London – responding to members’ needs at the time. Meetings can last for hours and involve complete strangers. I have often witnessed people walking in off the street, or straight from a hospital discharge for example. The meetings are informal and are conducted without set procedures or agendas. Meetings are reactive – responsive to personal stories, events or news – and topics of discussion are changeable and unpredictable. Official leadership or facilitation in meetings is rare and they are typically initiated by whoever wants to speak at the time. All are welcome, but there are rarely introductions – it is up to individuals to introduce themselves; sometimes members sit for hours together and never find out who one another are. There are no expectations of turn-taking, as in more conventional group therapy settings.

These arrangements have their difficulties – interlocutors sometimes express frustration about the lack of structure in meetings and, when there is a disagreement, there are no procedures in place to ask members to leave. But it is telling that these arrangements cannot just be read in the negative, as absences of positive qualities. Rather, they are consciously curated and challenge many assumptions about trusting therapeutic interactions needing to occur in contexts that are safe, ‘contained’, stable, orderly, predictable and familiar. Here, it is the very lack of order that is therapeutic, felt by participants to be empowering and healing, where rational institutional working had been found to be alienating, demoralising and stigmatising. Through certain ways of being-in-place, activists resist processes and relations that might in other contexts be seen as organisational virtues that define the gold standard of conventional healthcare – such as professionalism and expertise, impersonal institutionally defined roles and processes, and the standardised relationship, even the ‘routinised intimacy’, that characterises contemporary mental healthcare.^3^ Activists instead rely on spontaneity, mutual attentiveness, responsivity and informal group working.

Interlocutors speak about medical procedures associated with bureaucratic rationality, planning and accountability as countertherapeutic. During a meeting, one newcomer asked members of CMH why there were no meeting minutes, because then others could contribute remotely. One reply was, ‘If we took minutes, everything we say would be documented, get out of context and we would feel scrutinised’. Many interlocutors expressed a specific dislike of documentation (and particularly, being ‘written about’), especially those with experiences of being detained under the Mental Health Act (‘sectioned’) and/or claiming benefits (something that the majority of activists experience). Interlocutors described how it felt alienating and disempowering to not know what is written (or spoken) about them (i.e. in medical notes), particularly being unable to read or understand what the notes say when shorthand or medical terminology is used. Thus, meetings do not run according to agendas and the group do not write manifestos; rather, they operate on the principle that to have any ‘rules’ evokes those systems and modes of behaviour they specifically reject.

### How does this relate to the climate crisis?

For those for whom ‘therapeutic’ places provided by service providers are often harmful and exacerbate or elicit unwellness, making spaces according to these sensitivities requires flexibility. Therein lies Morton's ‘overhaul’; this rejection might be understood by psychiatrists and service managers in negative terms as an absence of organisation, or an inability to generate efficient ways of working. Understanding recovery collectively as activists do may even be read as a sign of dependence. Yet the climate crisis might suggest a different frame. A distaste for planning, organising and preparing reflects an awareness that these forms of organising (and the values and sensibilities that drive them) are discredited because the ecological crisis is driven by them. The production of pollutants on such a scale that they threaten life on earth demands industriousness, discipline and rational organisation on a huge scale. But as Bouton reminds us, we are all interdependent and interconnected and ‘all vulnerable’.^4^ Not acting in accordance with conventional psychotherapeutic thinking concerning relationships and ways of behaving enables mental health activists to have more control and ownership over their recovery, as does questioning the logic underpinning certain clinical ‘goals’ pertaining to health and wellness. Recovery for many survivor activists is relational, flexible and agentive and creating meeting spaces to behave in ‘disorderly’ ways is part of this process.

### Boundary objects

Psychiatric categories are an example of what Bowker & Star call ‘boundary objects’ – concepts that work across different institutional settings and contexts.^5^ Star & Griesemer define boundary objects as ‘objects which are both plastic enough to adapt to local needs and the constraints of the several parties employing them, yet robust enough to maintain a common identity across sites’.^6^ Boundary objects are terms that allow cooperation and communication between individuals (say, within an organisation) even if they do not necessarily agree with the precise meaning and definition of the terms. They have different meanings for different people. An example of such a term might be ‘recovery’: it is contested but also has a generalisable meaning in mental healthcare. Clinical terms designed to guide treatment decisions, for example, also guide access to benefits and relate to legal responsibility, capacity and disability. In this way psychopathology is tied to conformity and deviance as conceptualised in the context of the welfare state. But we can only take deviance as a sign of ill health and conformity as a marker of health if we think that society is more or less healthy. The climate crisis challenges that. Our collective inability to respond to emerging climate science looks compulsive and irrational, perhaps even delusional. It is functional people who produce and consume and thereby drive the production of greenhouse gases and undermine food security, while the economically inactive have the lightest carbon footprint.

### ‘Unemployment’ as mental illness or an enabler of social ‘goods’?

Survivor activists can be sceptical that good ‘health’ is so easily mapped onto capacity, function and ability to find and keep work. Many of CMH's campaigns revolve around the idea that people on benefits for mental illness are being pushed into work as part of new benefits changes and government targets related to financial management and (post)austerity measures and that this is harmful to claimants’ sense of autonomy and agency. CMH hosts film nights as opportunities to air concerns and grievances around such topics. One evening, Lissa, one of the founding members, stood in front of the small group of CMH members, therapy students and passers-by and announced that, ‘The government is trying to persuade us that unemployment is a mental illness. This driving force telling us we should all be in and doing productive work alters the sense of *who* and *what* we are’.

The discourse around ‘getting into work’ affects those in distress. Members discussed the connections between mental health services and the welfare system anxiously. Will, for example, a young activist in his 20s, feels guilty about his inability to work. He says he has never been in the position where ‘they thought enough of me to get to work’, even though he has tried. Will has spent most of his adult life living in supported accommodation and has been in hospital under various Mental Health Act sections. He was diagnosed with Asperger syndrome in school and had other mental health difficulties. He explains that he has undergone a work capability assessment and is waiting for the results. He feels as though he has to constantly justify why he has not worked and struggles with feeling illegitimate for never having had a job. At the job centre Will asked a receptionist whether he could just get ‘normal jobseeker's allowance’ instead of sickness or disability benefits. She was surprised and told him that he would receive more money by accepting illness benefits. Yet Will insisted on claiming jobseeker's allowance, lamenting that he ‘just wants to be like everyone else’.

At the film night, the group reflected on how people on benefits are treated with hostility and that they have the added disadvantage of having psychiatric diagnoses. ‘I don't want to go around being seen as the victim’, remarks one, ‘especially when we already have a self-blaming culture’. Julie raises a recent comment made by George Osborne about people on benefits lying in bed with their curtains drawn while others go out to work and that they remain closed when workers come home again.^7^ ‘We are trying to change this narrative,’ she says. Lissa adds that she cannot stand the perception that people on benefits for mental illness do not do much all day because they don't ‘work’. Her community psychiatric nurse (CPN) asked her what she ‘actually *does* all day’. So, she presented him with a list, ‘I get up early, check on my elderly mother, take my disabled sister to her hospital appointments, do her grocery shopping, call people up as part of my mental health support group mutual aid chats. Campaign for the end of workfare, write letters and articles. Lobby MPs, attend seminars in Westminster, draft responses and initiate public inquiries …’. Lissa's CPN was surprised, ‘It seems like you do more than me!’.

Julie explains that receiving benefits has meant that she gets to choose what she does with her life. She volunteers in the community, is a trained co-counsellor, runs literary events for mental health service survivors, supports benefit claimants with their claims and letters, and sanctions and lobbies Parliament to increase spending in mental health services in her free time. She supports as many friends and peers in mental distress as she can. She explains that, rather than running in ‘the rat race’, she has time to take action on behalf of those who are working. Employment caused her to have breakdowns. Implicit in her understanding is the idea that not working allows you to think, reflect, act collectively and undertake altruistic social ‘goods’; it gives you the time and energy to consider things that are bigger than you, to support others and gives you purpose and meaning.

## Conclusions

Wicked problems such as the climate crisis force us to rethink our understandings of what mental health is, how mental healthcare should be organised and what its goals should be. What we have learned from mental health service survivors is that, for them, it is healthy to challenge ideas about individual functioning and social responsibility. Resisting the pressure of working employment or assuming the role of a ‘productive’ member of a society by resisting workfare can be healthier for recovery than what is desired according to the psychiatric model of mental health. For activists, recovery outcomes are not connected to gaining employment. In fact, the push towards function via work/employment can exacerbate stress, feelings of stigma and of low self-worth. Therapeutic activities for activists instead involve creating environments for exploration in group settings, where the unpredictability and uncertainty of distress is given space. By interrogating the social causes of distress but refraining from seeking solutions to it, activists avoid attempting to fix or resolve problems and instead allow for ‘not knowing’. Thus, they suggest that the model of mental illness needs to be more flexible – it needs to allow for debate around what is classified as ‘healthy’ behaviour, to make room for dialogue and the open exploration of wicked problems, and to be reactive and responsive to the moment we are living in.

## Data Availability

The data are not publicly available because they contain information that could compromise the privacy of research participants.

## About the authors

**Neil Armstrong** is a stipendiary lecturer in anthropology at Magdalen College, University of Oxford, UK. **Keira Pratt-Boyden** is a PhD candidate in social anthropology at the School of Conservation and Anthropology, University of Kent, UK. She is also a part-time research assistant for the Anthropology of Open Dialogue (APOD) Project, SOAS University of London, UK.
